# Optoelectronic Properties of MAPbI_3_ Perovskite/Titanium Dioxide Heterostructures on Porous Silicon Substrates for Cyan Sensor Applications

**DOI:** 10.1186/s11671-015-1114-x

**Published:** 2015-10-16

**Authors:** Lung-Chien Chen, Chiao-Yu Weng

**Affiliations:** Department of Electro-optical Engineering, National Taipei University of Technology, 1, sec.3, Chung-Hsiao E. Rd., Taipei, 106 Taiwan

**Keywords:** Perovskite, TiO_2_, Porous silicon, Cyan sensors

## Abstract

This work elucidates the optoelectronic properties of graphene/methylammonium lead iodide (MAPbI_3_)/titanium dioxide (TiO_2_)/porous Si heterostructure diodes. The porous silicon substrates can accommodate more MAPbI_3_/TiO_2_ than the polished silicon substrate such that the MAPbI_3_/TiO_2_/porous Si substrate heterostructures have better optoelectronic properties. Photocurrents from 300 to 900 nm were measured. The photocurrent is high in two ranges of wavelength, which are 300–460 nm and 520–800 nm. The photocurrent plateau covers all visible light (360 to 780 nm) except for cyan between 460 and 520 nm. Therefore, the graphene/MAPbI_3_/TiO_2_/porous Si heterostructure can be utilized as cyan sensors.

## Background

Methylammonium lead iodide (CH_3_NH_3_PbI_3_ or MAPbI_3_) with the perovskite structure has potential optoelectronic applications, such as solar cells and light-emitting diodes, because of its direct band gap of 1.6 eV, low cost, and ease of production; it has therefore attracted substantial interest [1–5]. MAPbI_3_ perovskite-based solar cells with a power conversion efficiency of over 20 % have been successfully developed [6]. Such solar cells are promising because of their low cost, simplicity of fabrication, and absorption in the solar spectrum as well as balanced charge transport characteristics with long diffusion lengths [7, 8]. Light-emitting diodes that are based on halide perovskite have also been fabricated [9]. The green light-emitting device with the ITO/PEDOT:PSS/MAPbBr_3_/F8/Ca/Ag structure has a luminance of 364 cd/m^2^ at a current density of 123 mA/cm^2^ and external and internal quantum efficiencies of 0.1 and 0.4 %, respectively.

Last year, several works on perovskite-based photodetectors have been published [10, 11]. The absorption range of the MAPbI_3_ is very broad. The typical range is from 300 to 800 nm. However, excellent absorption of light cannot transform the absorbed energy into a photocurrent. Therefore, in this study, we developed the graphene/CH_3_NH_3_PbI_3_ (MAPbI_3_) perovskite/titanium dioxide (TiO_2_)/porous silicon substrate heterostructure diodes and studied their structure and optoelectronic properties.

## Methods

Single crystalline (100) p-type boron-doped Si substrates with a resistivity of 10 Ω cm were used in this study. Prior to processing, the wafers were cut into 1 × 1 cm^2^ and cleaned by ultrasonication in acetone, ethanol, and deionized water, consecutively. The silicon wafers then were immersed in dilute hydrogen fluoride (HF) solution to remove the native oxide layers, yielding a hydrogen-terminated surface. Next, the porous silicon structure was fabricated by metal-assisted chemical etching (MACE) in the freshly prepared dilute solution that contained both HF (48 %) and AgNO_3_ (0.02 mol/l) (1:1) with different etching times at room temperature. When the Si wafer was dipped into the etching solution, a silver nano-cluster layer was formed on its surface. The wafer was then put in a dilute HNO_3_ solution (50 wt%) to remove the Ag layer.

The TiO_2_ layer was coated onto the silicon substrate at a speed of 2000 rpm for 10 s and then annealed at 550 °C for 30 min. Subsequently, MAPbI_3_ perovskite precursor solution was coated onto the surface of the TiO_2_/silicon substrates using a spinner at a speed of 5000 rpm for 20 s. In this study, the perovskite layer was deposited by the solvent-engineering technology of 1.2 M Pbl_2_ and 1.2 M methylammonium iodide (MAI) in a cosolvent of dimethyl sulfoxide (DMSO) and γ-butyrolactone (GBL) (vol. ratio = 1:1) in a glove box filled with highly pure nitrogen. Then, the substrate was annealed at 100 °C for 10 min. The graphene layer was spin-coated using DMF-based graphene suspension (0.3 mg/ml) at 2000 rpm for 20 s. Finally, an indium contact (~2 μm) was evaporated onto the top of the graphene electron-spreading layer to complete the whole diode structure. The morphology and cross section of the resulting structures were examined using field emission scanning electron microscopy (FESEM). Photoluminescence (PL) was measured at room temperature. The excitation source for PL was a 405-nm diode laser. The electronic characteristics were measured using a Keithley 2420 programmable source meter.

## Results and Discussion

**Fig. 1:**
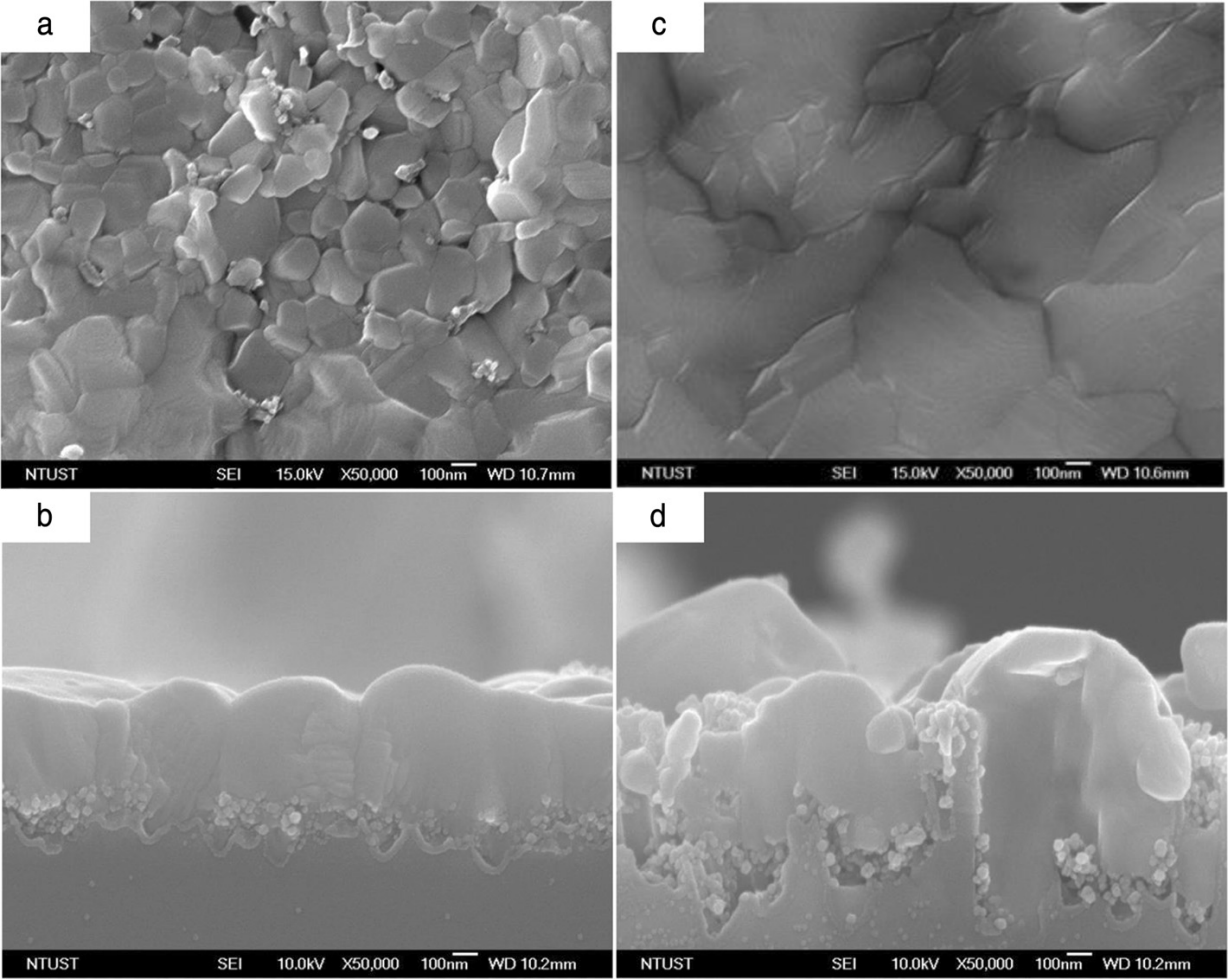
Schematic top view (**a**, **c**) and cross section (**b**, **d**) of MAPbI_3_/TiO_2_ on porous Si heterostructure

Figure 1 displays the top and cross-sectional FESEM images of the MAPbI_3_ perovskite/TiO_2_ on the porous Si substrates after etching for various times. Figure 1a, b presents the top and cross-sectional FESEM images of the MAPbI_3_ perovskite/TiO_2_ on the porous Si substrates that had been etched for 5 min. Figure 1c, d presents the top and cross-sectional FESEM images of the MAPbI_3_ perovskite/TiO_2_ on the porous Si substrates after etching for 10 min. The MAPbI_3_ perovskite was coated on the TiO_2_ layer, and the interface between the MAPbI_3_ perovskite and TiO_2_ layer formed the heterojunction. As presented in Fig. 1b, d, the MAPbI_3_ perovskite/TiO_2_ heterojunction penetrated into the porous silicon substrate. The thickness of the MAPbI_3_ perovskite films on the 5- and 10-min etched porous silicon substrates was around 500 nm.

**Fig. 2:**
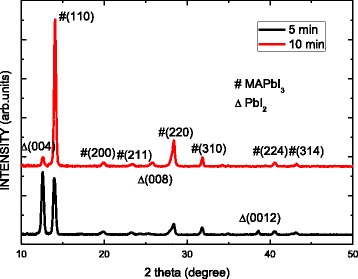
XRD patterns of MAPbI_3_ perovskite/TiO_2_ on porous Si substrate

Figure 2 shows the XRD patterns of the MAPbI_3_ perovskite/TiO_2_ on the porous Si substrate following etching for 5 and 10 min. The spectra include seven main peaks at 14.08°, 19.9°, 23.3°, 28.42°, 31.85°, 40.28°, and 43.21°, which correspond to the (110), (200), (211), (220), (310), (224), and (314) for the CH_3_NH_3_PbI_3_ perovskite, respectively. However, the PbI_2_ (004), (008), and (0012) peaks located at 12.58°, 25.81°, and 38.58°, respectively, can be observed in both samples. The coexistence of the two CH_3_NH_3_PbI_3_ and PbI_2_ phases can be observed in the MAPbI_3_ perovskite layers. This is due to the post annealing process leading to thermal decomposition of MAI and the formation of the PbI_2_ phase. Previous reports using transient photoluminescence exhibit the presence of the PbI_2_ in MAPbI_3_ active light harvesting layers that can enhance the carrier transportation to the electrode [12–14]. On the other hand, to further elucidate detailed structural information, the grain size G was calculated according to Scherrer’s equation [15]. The G grain sizes of the samples that were etched for 5 and 10 min are 22.2 and 29.5 nm, respectively. The quality of MAPbI_3_ on the porous silicon substrate with etching for 10 min is higher than that of the sample following etching for 5 min. The strongest signal from the samples with etching for 5 min is that of the (110) plane. However, the most intense signal of the samples that were etched for 10 min is that of the (101) plane. The morphology of the Si substrate influences the formation of the crystalline MAPbI_3_.

**Fig. 3:**
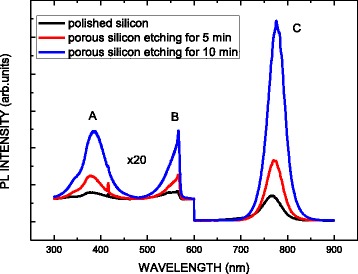
RT PL spectrum of graphene/MAPbI_3_/TiO_2_/porous Si heterostructure

Figure 3 shows the room-temperature photoluminescence (RT PL) spectrum of the MAPbI_3_/TiO_2_/porous Si heterostructure. From all samples, the dominant peak at 1.6 eV (776 nm), labeled C, corresponds to the optical band gap of the MAPbI_3_ films, which have a direct band gap that can be attributed to the recombination of the near band-to-band (B-B) [1, 16]. Other than peak C, two weak peaks, A and B, are observed. The A peak at 382 nm corresponds to the recombination of the B-B of TiO_2_ [17]. Peak B at 566 nm is associated with the emission from defects in TiO_2_ [17–19]. The PL intensity increases with the etching time of the silicon substrate. That may be attributed to the amount of the TiO_2_ and MAPbI_3_ penetrated into the porous silicon substrates to fill in the porous increases due to the dimension increase of the porous when etching time increases.

**Fig. 4:**
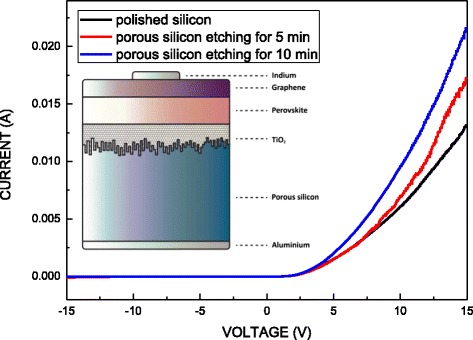
I-V characteristics of graphene/MAPbI_3_/TiO_2_/Si heterostructure in darkness. *Inset* schematically depicts the graphene/MAPbI_3_/TiO_2_/Si heterostructure diode

**Fig. 5:**
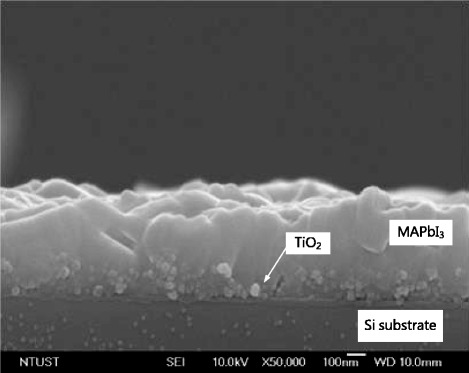
Cross-sectional FESEM image of the MAPbI_3_/TiO_2_ on polished silicon substrate

Figure 4 plots the I-V characteristics of the graphene/MAPbI_3_/TiO_2_/porous Si heterostructure diode in darkness. The turn-on voltage of the graphene/MAPbI_3_/TiO_2_/porous Si heterostructure diode is approximately 2.5 V, and its breakdown voltage is over 15 V. The inset schematically depicts the graphene/MAPbI_3_/TiO_2_/porous Si heterostructure diode. The graphene/MAPbI_3_/TiO_2_/porous Si that was etched for 10 min exhibited the lowest series resistance, because it had the highest effective contact area of porous silicon and accommodated the largest MAPbI_3_/TiO_2_ bulk junction, relative to the polished silicon substrate, as shown in Figs. 1b, d and 5. As mentioned above, the purity and grain size of MAPbI_3_ on the porous silicon substrate with etching for 10 min is higher than that of the sample with etching for 5 min. It is believed that the TiO_2_ nanoparticle deposited on the substrate etched for 10 min can provide more surface area and good contact, leading to less MAI loss and better crystal quality. The morphology of the Si substrate influences the formation of the crystalline MAPbI_3_ and optoelectronic characteristics of MAPbI_3_-based devices.

**Fig. 6:**
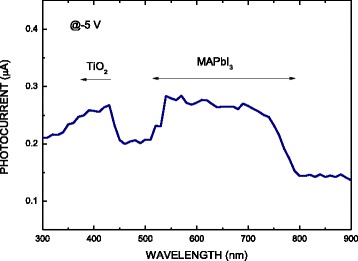
Photocurrent as a function of wavelength of incident light for a graphene/MAPbI_3_/TiO_2_/porous Si heterostructure at a bias of 5 V

Figure 6 plots the photocurrent as a function of the wavelength of incident light for a graphene/MAPbI_3_/TiO_2_/porous Si heterostructure at a reverse bias of 5 V. The photocurrent is high in two ranges of wavelength from 300 to 450 nm and from 520 to 780 nm. The former corresponds to TiO_2_ and the latter corresponds to the MAPbI_3_. Two photocurrents tail off at wavelengths of 460 and 780 nm. As compared to the pure TiO_2_ with an absorption edge of 400 nm, the tailing off at 460 nm suggests the presence of traps in the band gap of the TiO_2_ that are generated by the presence of impurities from MAPbI_3_ [20]. The tailing off at 770 nm suggests the band gap 1.6 eV of the MAPbI_3_. The photocurrent plateau covers all visible wavelengths (360 to 780 nm) except for those of cyan from 460 to 520 nm. Therefore, the graphene/MAPbI_3_/TiO_2_/porous Si heterostructure can be used in a cyan sensor.

## Conclusions

The optoelectronic characteristics of graphene/MAPbI_3_/TiO_2_/Si heterostructure diodes were investigated. As the etching time of the silicon substrate increased, the PL intensity increased, because more TiO_2_ and MAPbI_3_ penetrated into the porous silicon substrates, because they accommodated more MAPbI_3_/TiO_2_. The MAPbI_3_/TiO_2_ on the porous silicon substrate increased the effective contact area of the heterostructure and reduced its series resistance. The photocurrent plateau covered all visible wavelengths (360 to 780 nm), except for those of cyan from 460 to 520 nm. Therefore, as shown in Fig. 6, the graphene/MAPbI_3_/TiO_2_/porous Si heterostructure can not only be utilized in a cyan sensor but also for near-IR light (780–900 nm or more) applications.
